# Correction: Diet quality in young adulthood and sleep at midlife: a prospective analysis in the Bogalusa Heart Study

**DOI:** 10.1186/s12937-024-01055-8

**Published:** 2024-12-02

**Authors:** Kaitlin S. Potts, Jeanette Gustat, Maeve E. Wallace, Sylvia H. Ley, Lu Qi, Lydia A. Bazzano

**Affiliations:** 1 Department of Epidemiology, Tulane University School of Public Health and Tropical Medicine, New Orleans, LA USA; 2 Division of Sleep and Circadian Disorders, Division of Sleep Medicine, Brigham and Women’s Hospital, Harvard Medical School, Boston, MA USA; 3 Department of Social, Behavioral and Population Sciences, Tulane School of Public Health and Tropical Medicine, New Orleans, LA USA; 4 Department of Nutrition, Harvard T.H. Chan School of Public Health, Boston, MA USA

**Correction: Nutr J 23**, ** 128 (2024)**

10.1186/s12937-024-01033-0

Following publication of the original article [1], the author reported that there is a typographical error in the first sentence of the Methods section.

The phrase “in a biracial (Black/**While)** population” should say “in a biracial (Black/**White**) population”.

The original article has been updated.
