# Supplementary material for: Diet quality in young adulthood and sleep at midlife: a prospective analysis in the Bogalusa Heart Study
Source: Nutr J. 2024 Oct 22;23:128. doi: 10.1186/s12937-024-01033-0 (PMC11494891; doi:10.1186/s12937-024-01033-0)
Supplement: Supplementary file 1 — Additional File 1: Figure S1: Participant flowchart; Table S1. Dietary patterns’ components and scoring; Table S2: Women’s Health Initiative Insomnia Rating Scale (WHIIRS); Table S3: Berlin questionnaire for sleep apnea risk; Table S4: Description of dietary patterns; Table S5: Baseline characteristics of participants by sleep outcomes at follow-up; Table S6: Comparison of baseline characteristics of those included versus those lost to follow-up or excluded; Table S7: Results of interaction analyses. P-values for product-terms between dietary pattern variables and sex, race/ethnicity, and education level; Table S8: Stratified analyses not reported in main tables, where one or more interaction terms were statistically significant. Risk ratios for sleep outcomes by baseline dietary pattern scores stratified by sex or race; Table S9: Sensitivity analysis adjusting for sleep duration: Risk ratios for high insomnia symptoms at follow-up by baseline dietary pattern scores (n-571); Table S10: Sensitivity analysis removing BMI from models for sleep apnea: Risk ratios for high sleep apnea risk at follow-up by baseline dietary pattern scores; Table S11: Risk ratios for sleep outcomes, components of the Berlin Questionnaire, at follow-up by baseline dietary pattern scores; Table S12: Risk ratios for being healthy on components of the healthy sleep pattern, at follow-up by baseline dietary pattern scores [file 12937_2024_1033_MOESM1_ESM.docx]

**Supplementary Material**

**List of supplemental figures and tables**

Figure S1. Participant flowchart.

Table S1. Dietary patterns’ components and scoring.

Table S2. Women’s Health Initiative Insomnia Rating Scale (WHIIRS).

Table S3. Berlin questionnaire for sleep apnea risk.

Table S4. Description of dietary patterns.

Table S5. Baseline characteristics of participants by sleep outcomes at follow-up.

Table S6. Comparison of baseline characteristics of those included versus those lost to follow-up or excluded.

Table S7. Results of interaction analyses. P-values for product-terms between dietary pattern variables and sex, race/ethnicity, and education level.

Table S8. Stratified analyses not reported in main tables, where one or more interaction terms were statistically significant. Risk ratios for sleep outcomes by baseline dietary pattern scores stratified by sex or race.

Table S9. Sensitivity analysis adjusting for sleep duration: Risk ratios for high insomnia symptoms at follow-up by baseline dietary pattern scores (n-571).

Table S10. Sensitivity analysis removing BMI from models for sleep apnea: Risk ratios for high sleep apnea risk at follow-up by baseline dietary pattern scores.

Table S11. Risk ratios for sleep outcomes, components of the Berlin Questionnaire, at follow-up by baseline dietary pattern scores.

Table S12. Risk ratios for being healthy on components of the healthy sleep pattern, at follow-up by baseline dietary pattern scores.

Figure S1. Participant flowchart.


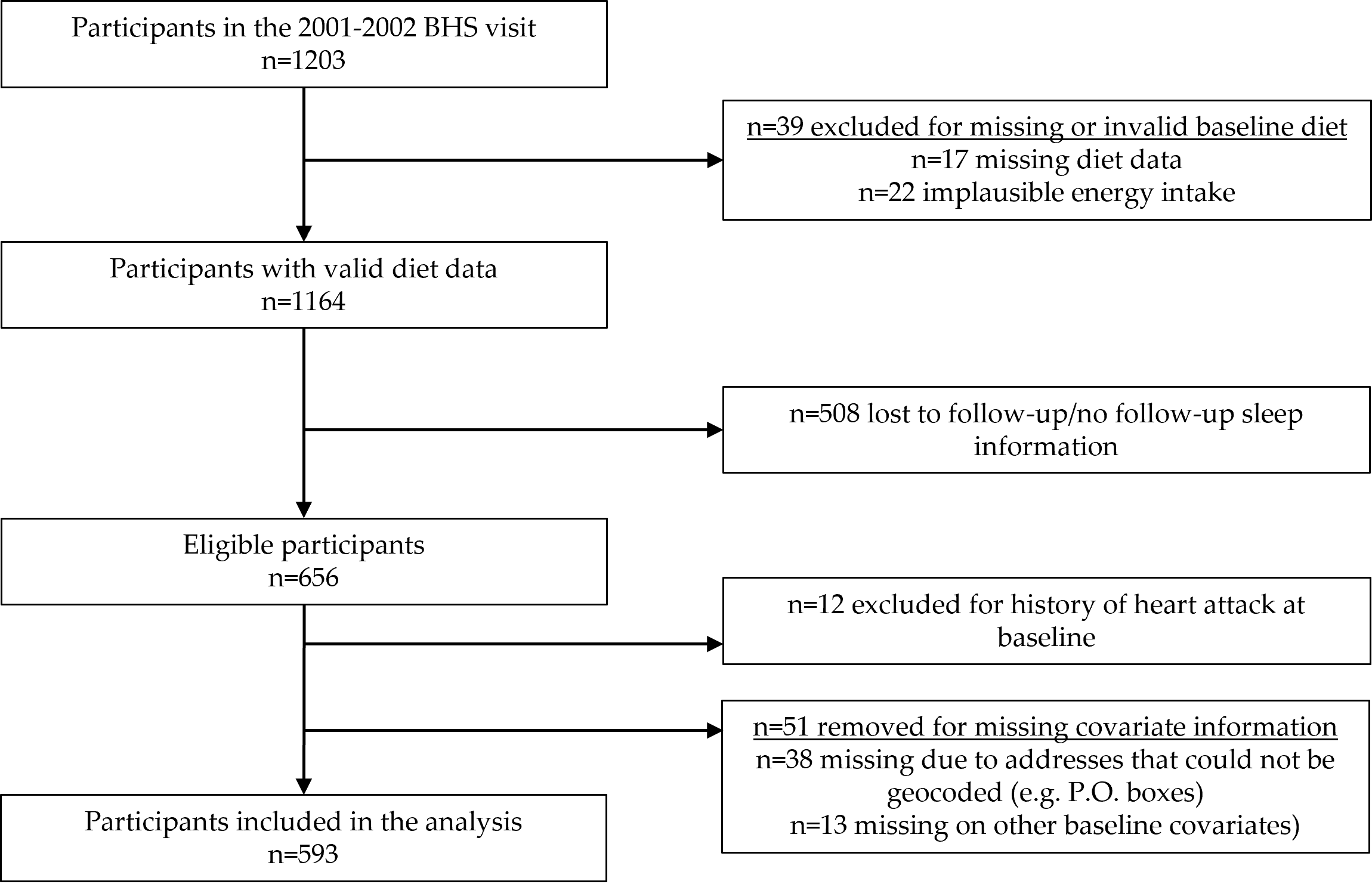


Table S1. Dietary patterns’ components and scoring.

|  | **AHEI-2010** | | **HEI-2015** | | **aMed** | |
| --- | --- | --- | --- | --- | --- | --- |
| **Summary** | 11 components  Total score: 0-110 | Components score 0-10 between the criteria for min and max score. | 13 components  Total score: 0-100 | Components score 0-5 or 0-10 between the criteria for min and max score. | 9 components  Total score: 0-9 | Components score 0-1 based on sex-specific medians |
|  | **Component** | **Scoring** | **Component** | **Scoring** | **Component** | **Scoring** |
| Higher intake 🡪 higher score | 1. Fruits | 0: 0 servings  10: ≥ 4 servings | 1. Total fruits (including 100% juice) | 0: no fruit  5: ≥ 0.8 cup per 1000 kcal | 1. Fruit | 0: < median  1: > median |
|  | *1 serving =1 medium piece or 0.5 cup berries* | |  |  |  |  |
|  | 2. Vegetables (not potatoes) | 0: 0 servings  10: ≥ 5 servings | 2. Whole fruits | 0: no whole fruit  5: ≥ 0.4 cup per 1000 kcal | 2. Vegetables (not potatoes) | 0: < median  1: > median |
|  | *1 serving=0.5 cups vegetables or 1 cup greens* | |  |  |  |  |
|  | 3. Nuts and legumes | 0: 0 servings  10: ≥ 1 servings | 3. Total vegetables (incl. legumes) | 0: no vegetables  5: ≥ 1.1 cup per 1000 kcal | 3. Nuts | 0: < median  1: > median |
|  | *1 serving=1 oz nuts or 1 tbsp nut butter* | |  |  |  |  |
|  | 4. Whole grains | 0: 0 grams  10: men 🡪 ≥ 90 grams  women 🡪 ≥ 75 grams | 4. Greens and beans | 0: no dark greens or legumes  5: ≥ 0.2 cup per 1000 kcal | 4. Legumes | 0: < median  1: > median |
|  | 5. Long chain (n-3) fats (EPA + DHA) | 0: 0 mg  10: ≥ 250 mg (~2-4 servings of fish per week) | 5. Whole grains | 0: no whole grains  10: ≥ 1.5 oz per 1000 kcal | 5. Whole grains | 0: < median  1: > median |
|  | 6. PUFAs | 0: ≤ 2% of energy  10: ≥ 10% of energy | 6. Dairy | 0: no diary  10: ≥ 1.3 cup per 1000 kcal | 6. Fish | 0: < median  1: > median |
|  |  |  | 7. Total protein foods | 0: no protein foods  5: ≥ 2.5 oz per 1000 kcal | 7. Fatty acids ratio: (MUFAs/ SFAs) | 0: < median  1: > median |
|  |  |  | 8. Seafood and plant proteins | 0: no seafood or plant proteins  5: ≥ 0.8 oz per 1000 kcal |  |  |
|  |  |  | 9. Fatty acids ratio | 0: (PUFAs + MUFAs) / SFAs ≤ 1.2  10: (PUFAs + MUFAs) / SFAs ≥ 2.5 |  |  |
| Lower intake 🡪 higher score | 7. Sugar sweetened beverages and fruit juice | 0: 0  10: ≥ 1 serving | 10. Refined grains | 0: ≥ 4.3 oz per 1000 kcal  10: ≤ 1.8 oz per 1000 kcal | 8. Red and processed meats | 0: > median  1: < median |
|  | *1 serving=8 oz* | |  |  |  |  |
|  | 8. Red and processed meats | 0: ≥ 1.5 servings  10: 0 servings | 11. Added sugars | 0: ≥ 26% of energy  10: ≤ 6.5% of energy |  |  |
|  | *1 serving=4 oz unprocessed meat or 1.5 oz processed meat* | |  |  |  |  |
|  |  |  | 12. Saturated fats | 0: ≥ 16% of energy  10: ≤ 8% of energy |  |  |
|  | 10. Sodium | 0: highest decile  10: lowest decile | 13. Sodium | 0: ≥ 2.0 grams per 1000 kcal  10: ≤1.1 grams per 1000 kcal |  |  |
| Moderate intake 🡪 highest score | 11. Alcohol | 0: men 🡪 ≥ 3.5 drinks  women 🡪 ≥ 2.5 drinks  10: men 🡪 0.5-2.0 drinks  women 🡪 0.5-1.5  drinks |  |  | 9. Alcohol | 0: men 🡪 <1 or  >2 drinks  women 🡪 <0.5  or > 1.5  drinks  1: men 🡪 1 to 2  drinks  women 🡪 0.5  to 1.5 drinks |

All scores based on intake per day.

PUFA: polyunsaturated fatty acids.

MUFA: monounsaturated fatty acids.

SFA: saturated fatty acids.

Table S2. Women’s Health Initiative Insomnia Rating Scale (WHIIRS). Each question scores 1-5 points as shown. The summed individual scores give the total WHIIRS score where >9 indicates high risk for insomnia/high insomnia symptoms.

| **Question (In the last 4 weeks…)** | **Score) Response options** |
| --- | --- |
| 1. Did you have trouble falling asleep? | 1. “no, not in the past 4 weeks” 2. “yes, less than once a week” 3. “yes, 1 or 2 times a week” 4. “yes, 3 or 4 times a week” 5. “yes, 5 or more times a week” |
| 1. Did you wake up several times at night? |  |
| 1. Did you wake up earlier than you planned to? |  |
| 1. Did you have trouble getting back to sleep after you woke up too early? |  |
| 1. Overall, was your typical night’s sleep during the last 4 weeks: | 1. “very sound or restful” 2. “sound or restful” 3. “average quality” 4. “restless” 5. “very restless” |

Table S3. Berlin questionnaire for sleep apnea risk. Respondent considered high risk for sleep apnea if positive on two of the three components.

| **Category** | **Question (In the last 4 weeks)** | **Response options** |
| --- | --- | --- |
| Category 1, **Snoring**: this category is positive if the sum of the 5 items is 2 or more | 1. Have you snored? | 1. Yes (1 point) 2. No 3. Don’t know |
|  | 1. Your snoring is: | 1. Slightly louder than breathing 2. As loud as talking (1 point) 3. Louder than talking (1 point) 4. Very loud-can be heard in adjacent rooms (1 point) |
|  | 1. How often do you snore? | 1. Almost every day (1 point) 2. 3-4 times/week (1 point) 3. 1-2 times/week 4. 1-2 times/month 5. Never |
|  | 1. Does your snoring bother other people? | 1. Yes (1 point) 2. No 3. Don’t know |
|  | 1. Has anyone ever noticed you stop breathing during your sleep? | 1. Almost every day (2 points) 2. 3-4 times/week (2 points) 3. 1-2 times/week 4. 1-2 times/month 5. Never |
| Category 2, **Sleepiness**: this category is positive if the sum is 2 or more | 1. How often do you feel tired or fatigued after you sleep? | 1. Almost every day (1 point) 2. 3-4 times/week (1 point) 3. 1-2 times/week 4. 1-2 times/month 5. Never |
|  | 1. During your waking time, do you feel tired, fatigued, or not up to par? | 1. Almost every day (1 point) 2. 3-4 times/week (1 point) 3. 1-2 times/week 4. 1-2 times/month 5. Never |
|  | 1. Have you ever nodded off or fallen asleep while driving? | Yes (1 point)  No  Don’t know |
| Category 3, **Obesity/ Hypertension**: this category is positive if score is 1 or more | 1. BMI > 30 kg/m^2^? | Yes (1 point)  No |
|  | 1. High blood pressure? | Yes (1 point)  No |

Table S4. Description of dietary patterns.

|  | **Total sample** | | **Q1** | **Q2** | **Q3** | **Q4** | **Q5** |
| --- | --- | --- | --- | --- | --- | --- | --- |
|  | mean ± SD | median (min, max) | n (median) | n (median) | n (median) | n (median) | n (median) |
| AHEI-2010 | 37.96 ± 7.81 | 37.20 (18.23, 68.53) | 127 (28.66) | 116 (33.93) | 129 (37.57) | 112 (42.41) | 109 (48.99) |
| HEI-2015 | 58.16 ± 9.42 | 57.73 (30.15, 87.19) | 111 (45.89) | 116 (52.62) | 130 (57.47) | 114 (62.58) | 122 (69.98) |
| aMed | 4.33 ± 1.76 | 4.00 (0.00, 8.00) | 98 (2.00) | 90 (3.00) | 121 (4.00) | 127 (5.00) | 157 (6.00) |
| AHEI: Alternate Healthy Eating Index. HEI: Healthy Eating Index. aMed: alternate Mediterranean. | | | | | | | |

Table S5. Baseline characteristics of participants by sleep outcomes at follow-up.

|  | **Insomnia risk ^a^** | | | **Sleep apnea risk ^a^** | | | **Healthy sleep pattern ^a^** | | |
| --- | --- | --- | --- | --- | --- | --- | --- | --- | --- |
|  | **Low** | **High** | **p-value ^b^** | **Low** | **High** | **p-value ^b^** | **No** | **Yes** | **p-value ^b^** |
|  | n=325 | n=268 |  | n=351 | n=242 |  | n=454 | n=133 |  |
| ***Demographic characteristics at baseline (2001-2002)*** | | | | | | | | | |
| Age in years | 36.23 ±4.59 | 36.41 ± 4.20 | 0.632 | 36.16 ± 4.43 | 36.53 ± 4.39 | 0.322 | 36.20 ± 4.42 | 36.70 ± 4.42 | 0.256 |
| Male (%) | 36.69 | 32.09 | 0.055 | 33.05 | 40.91 | 0.050 | 36.56 | 35.34 | 0.796 |
| Black persons (%) | 32.62 | 26.87 | 0.1284 | 28.77 | 31.82 | 0.427 | 30.62 | 27.82 | 0.536 |
| Education, high school or less (%) | 39.08 | 39.55 | 0.906 | 40.74 | 37.19 | 0.384 | 37.00 | 46.62 | 0.046 |
| Income at baseline |  |  |  |  |  |  |  |  |  |
| <$15,000 | 22.77 | 28.36 | 0.3169 | 25.93 | 24.38 | 0.622 | 25.33 | 24.06 | 0.454 |
| $15,000-$30,000 | 22.15 | 19.03 |  | 19.09 | 23.14 |  | 20.48 | 21.80 |  |
| $30,000-$45,000 | 12.00 | 13.81 |  | 13.68 | 11.57 |  | 13.88 | 9.02 |  |
| >$45,000 | 43.08 | 38.81 |  | 41.31 | 40.91 |  | 40.31 | 45.11 |  |
| Employed (%) | 76.92 | 80.60 | 0.278 | 76.92 | 80.99 | 0.235 | 79.96 | 74.44 | 0.172 |
| Has health insurance (%) | 68.31 | 66.04 | 0.559 | 67.24 | 67.36 | 0.976 | 67.40 | 66.17 | 0.790 |
| Household size | 3.40 ± 1.28 | 3.55 ± 1.33 | 0.177 | 3.40 ± 1.29 | 3.56 ± 1.33 | 0.149 | 3.52 ± 1.30 | 3.32 ± 1.31 | 0.136 |
| Lives with spouse (%) | 63.38 | 60.07 | 0.409 | 62.39 | 61.16 | 0.761 | 62.56 | 60.15 | 0.615 |
| Children in house (%) | 72.92 | 74.63 | 0.639 | 71.79 | 76.45 | 0.206 | 74.45 | 70.68 | 0.385 |
| ***Neighborhood characteristics (census tract level, 2000 Decennial Census)*** | | | | | | | | | |
| Total population | 4986.12 ± 1781.95 | 5172.60 ± 1924.55 | 0.222 | 5159.57 ± 1808.52 | 4941.06 ± 1901.35 | 0.157 | 4977.44 ± 1785.81 | 5398.08 ± 2057.26 | 0.034 |
| % persons in poverty | 23.61 ± 8.71 | 23.71 ± 8.55 | 0.892 | 23.38 ± 8.80 | 24.05 ± 8.39 | 0.352 | 23.77 ± 8.61 | 23.12 ± 8.75 | 0.447 |
| Median household income | 26732.42 ± 10126.09 | 26596.21 ± 10736.00 | 0.874 | 27000.26 ± 10638.15 | 26193.10 ± 10040.76 | 0.353 | 26537.12 ± 10124.61 | 27305.68 ± 11444.10 | 0.455 |
| % households w/no vehicle | 11.95 ± 6.97 | 11.46 ± 6.83 | 0.390 | 11.53 ± 6.90 | 12.03 ± 6.91 | 0.383 | 11.98 ± 6.93 | 10.86 ± 6.72 | 0.100 |
| Index of Concentration at the Extremes | -0.13 ± 0.20 | -0.12 ± 0.19 | 0.710 | -0.12 ± 0.19 | -0.13 ±0.19 | 0.616 | -0.13 ± 0.19 | -0.12 ± 0.19 | 0.427 |
| ***Health and lifestyle factors at baseline (2001-2002)*** | | | | | | | | | |
| Smoking status (%) |  |  |  |  |  |  |  |  |  |
| Never | 63.69 | 56.34 | 0.190 | 63.53 | 55.79 | 0.041 | 58.59 | 66.92 | 0.049 |
| Former | 11.69 | 13.81 |  | 9.97 | 16.53 |  | 14.54 | 6.77 |  |
| Current | 24.62 | 29.85 |  | 26.50 | 27.69 |  | 26.87 | 26.32 |  |
| Current alcohol use (%) |  |  |  |  |  |  |  |  |  |
| Non-drinker | 36.62 | 33.96 | 0.743 | 34.19 | 37.19 | 0.684 | 35.90 | 34.59 | 0.715 |
| Occasional drinker | 37.85 | 40.67 |  | 40.46 | 37.19 |  | 38.33 | 42.11 |  |
| Regular drinker | 25.54 | 25.37 |  | 25.36 | 25.62 |  | 25.77 | 23.31 |  |
| Total energy intake, kcal/d | 2008.50 ± 796.71 | 2002.35 ±720.94 | 0.922 | 1969.75 ± 792.06 | 2057.90 ± 716.58 | 0.167 | 2034.61 ± 772.61 | 1893.77 ± 679.61 | 0.058 |
| Caffeine intake, mg/d | 91.25 ± 65.95 | 107.03 ± 68.64 | 0.005 | 96.18 ± 68.67 | 101.58 ± 65.98 | 0.340 | 97.26 ± 17.08 | 102.57 ± 70.97 | 0.425 |
| Physically active at work (%) | 38.15 | 41.42 | 0.419 | 39.89 | 39.26 | 0.878 | 40.31 | 37.59 | 0.574 |
| Physically active not at work (%) | 33.23 | 27.99 | 0.169 | 32.48 | 28.51 | 0.304 | 30.18 | 32.33 | 0.636 |
| Depressive symptoms (%) | 26.77 | 35.82 | 0.018 | 30.20 | 31.82 | 0.675 | 31.94 | 26.32 | 0.216 |
| CES-D score | 11.28 ± 8.05 | 14.23 ± 9.62 | <0.0001 | 12.49 ± 8.88 | 12.79 ± 8.97 | 0.681 | 13.06 ± 8.97 | 10.80 ± 8.21 | 0.010 |
| Body mass index, kg/m^2^ | 28.90 ± 7.01 | 29.67 ± 7.56 | 0.200 | 27.75 ± 7.03 | 31.42 ± 7.07 | <0.0001 | 29.74 ± 7.24 | 27.26 ± 6.92 | 0.001 |
| Obesity (%) | 36.92 | 42.91 | 0.138 | 28.77 | 55.37 | <0.0001 | 43.17 | 25.56 | 0.0003 |
| Waist circumference, cm | 92.11 ± 16.49 | 92.79 ± 17.68 | 0.630 | 88.64 ± 16.52 | 97.89 ± 16.28 | <0.0001 | 93.75 ± 17.08 | 87.29 ± 15.89 | 0.0001 |
| ***Dietary patterns*** | | | | | | | | |  |
| AHEI-2010 | 38.92 ± 7.73 | 36.80 ± 7.78 | 0.001 | 38.35 ± 7.78 | 37.39 ± 7.85 | 0.138 | 37.87 ± 7.82 | 38.23 ± 7.74 | 0.641 |
| HEI-2015 | 59.20 ± 9.80 | 56.90 ± 8.80 | 0.003 | 58.59 ± 10.00 | 57.54 ± 8.50 | 0.168 | 58.16 ± 9.43 | 57.97 ± 9.55 | 0.838 |
| aMed | 4.38 ± 1.73 | 4.27 ± 1.79 | 0.425 | 4.31 ± 1.80 | 4.36 ± 1.70 | 0.754 | 4.38 ± 1.72 | 4.15 ± 1.90 | 0.194 |

^a.^ Frequency (%) or mean ±SD among column total (total in quintile).

^b.^ p-value from t-test for continuous covariates and from Pearson chi-squared test for independence for categorical covariates.

^*^ n=589 for Healthy Sleep Pattern

CES-D: Center for Epidemiologic Studies Depression Scale. AHEI: Alternative Healthy Eating Index. HEI: Healthy Eating Index. aMed: Alternate Mediterranean dietary pattern.

Physically active: self-rating of 4 or 5 (active or very active) on 5-point scale.

Index of Concentration at the Extremes: ([(number of White householders with >$100,000 annual income)-(number of Black householders with <$25,000 annual income)] / total households reporting income)

Occasional drinker: less than once a week; Regular drinker: once or twice a week, or more.

Depressive symptoms defined as CES-D ≥ 16.

Obesity defined as BMI ≥ 30 kg/m^2^.

High risk for insomnia: score >9 on the Women’s Health Initiative Insomnia Rating Scale.

High risk for sleep apnea: positive on two of three categories on the Berlin questionnaire.

Healthy sleep pattern: morning or more morning-than-evening chronotype, average sleep duration ≥7 and <9 hours, insomnia symptoms <1-2 times/wk, snoring is quiet and infrequent (≤1-2 times/wk), and no excessive daytime sleepiness.

Table S6. Comparison of baseline characteristics of those included versus those lost to follow-up or excluded.

|  | **Included in the analysis ^a^** | **Excluded or lost to follow-up ^a^** | **p-value ^b^** |
| --- | --- | --- | --- |
|  | n=593 | n=610 |  |
| ***Demographic characteristics at baseline (2001-2002)*** | | | |
| Age in years | 36.31 ± 4.41 | 36.29 ± 4.52 | 0.938 |
| Male (%) | 36.26 | 49.75 (n=605) | <0.0001 |
| Black persons (%) | 30.02 | 30.33 | 0.907 |
| Education, high school or less (%) | 39.29 | 39.04 (n=607) | 0.930 |
| Income at baseline |  | (n=602) |  |
| <$15,000 | 25.30 | 26.91 | 0.298 |
| $15,000-$30,000 | 20.74 | 20.10 |  |
| $30,000-$45,000 | 12.82 | 15.95 |  |
| >$45,000 | 41.15 | 37.04 |  |
| Employed (%) | 78.58 | 74.79 (n=607) | 0.121 |
| Has health insurance (%) | 67.28 | 67.55 (n=607) | 0.923 |
| Household size | 3.47 ± 1.31 | 3.47 ± 1.48 (n=607) | 0.977 |
| Lives with spouse (%) | 61.89 | 59.47 (n=607) | 0.392 |
| Children in house (%) | 73.69 | 66.56 (n=607) | 0.007 |
| ***Neighborhood characteristics (census tract level, 2000 Decennial Census)*** | | | |
| Total population | 5070.39 ± 1848.51 | 4995.66 ± 1757.87 (n=540) | 0.487 |
| % of persons in poverty | 23.66 ± 8.63 | 23.04 ± 8.95 (n=540) | 0.242 |
| Median household income | 26670.86 ± 10397.49 | 27773.69 ± 12257.14 (n=540) | 0.104 |
| % of households with no vehicle | 11.73 ± 6.90 | 11.61 ± 6.90 (n=540) | 0.771 |
| Index of Concentration at the Extremes | -0.13 ± 0.19 | -0.12 ± 0.20 (n=540) | 0.422 |
| ***Health and lifestyle factors at baseline (2001-2002)*** | | | |
| Smoking status (%) |  | (n=606) |  |
| Never | 60.37 | 53.30 | 0.007 |
| Former | 12.65 | 11.22 |  |
| Current | 26.98 | 35.48 |  |
| Current alcohol use (%) |  | (n=607) |  |
| Non-drinker | 35.41 | 34.93 | 0.129 |
| Occasional drinker | 39.12 | 34.76 |  |
| Regular drinker | 25.46 | 30.31 |  |
| Total energy intake, kcal/d | 2005.72 ±762.77 | 2091.67 ± 1019.29 (n=580) | 0.103 |
| Caffeine intake, mg/d | 98.38 ± 67.58 | 102.85 ± 72.41 (n=580) | 0.275 |
| Physically active at work (%) | 39.63 | 38.49 (n=608) | 0.685 |
| Physically active not at work (%) | 30.86 | 28.62 (n=608) | 0.395 |
| Depressive symptoms (%) | 30.86 | 35.42 (n=607) | 0.094 |
| CES-D score | 12.61 ± 8.91 | 13.67 ± 9.89 (n=607) | 0.052 |
| Body mass index, kg/m^2^ | 29.25 ± 7.27 | 29.91 ± 7.27 | 0.117 |
| Obesity (%) | 39.63 | 41.64 | 0.478 |
| Waist circumference, cm | 92.42 ± 17.03 | 95.62 ± 18.16 | 0.002 |
| ***Dietary pattern scores at baseline (2001-2001)*** | | | |
| AHEI-2010 | 37.96 ± 7.81 | 38.92 ± 8.19 (n=577) | 0.040 |
| HEI-2015 | 58.16 ± 9.42 | 57.78 ± 9.79 (n=577) | 0.496 |
| aMed | 4.33 ± 1.76 | 4.31 ± 1.76 (n=577) | 0.831 |
| ***Sleep outcomes at follow-up (2013-2016)*** | | | |
| High risk for insomnia (%) | 45.19 | 49.45 (n=91) | 0.448 |
| High risk for sleep apnea (%) | 40.81 | 51.65 (n=91) | 0.051 |
| Healthy sleep pattern (%) ^*^ | 22.66 | 17.98 (n=89) | 0.321 |

^a.^ Frequency (%) or mean ±SD among total sample.

^b.^ p-value from t-test for continuous covariates and from Pearson chi-squared test for independence for categorical covariates.

^*^ n=589 for Healthy Sleep Pattern

CES-D: Center for Epidemiologic Studies Depression Scale. AHEI: Alternative Healthy Eating Index. HEI: Healthy Eating Index. aMed: Alternate Mediterranean dietary pattern.

Physically active: self-rating of 4 or 5 (active or very active) on 5-point scale.

Index of Concentration at the Extremes: ([(number of White householders with >$100,000 annual income)-(number of Black householders with <$25,000 annual income)] / total households reporting income)

Occasional drinker: less than once a week; Regular drinker: once or twice a week, or more.

Depressive symptoms defined as CES-D ≥ 16.

Obesity defined as BMI ≥ 30 kg/m^2^.

High risk for insomnia: score >9 on the Women’s Health Initiative Insomnia Rating Scale.

High risk for sleep apnea: positive on two of three categories on the Berlin questionnaire.

Healthy sleep pattern: morning or more morning-than-evening chronotype, average sleep duration ≥7 and <9 hours, insomnia symptoms <1-2 times/wk, snoring is quiet and infrequent (≤1-2 times/wk), and no excessive daytime sleepiness.

Table S7. Results of interaction analyses. P-values for product-terms between dietary pattern variables and sex, race/ethnicity, and education level.

|  |  | p-value for product of effect modifier * **dietary pattern total score** | p-value for product of effect modifier * **Q5 of dietary pattern** | p-value of effect modifier * **dietary pattern trend variable** |
| --- | --- | --- | --- | --- |
| **Effect modifier: Sex** | | | | |
| **Dietary pattern** | **Outcome** |  |  |  |
| **AHEI-2010** | Insomnia risk | 0.302 | 0.176 | 0.077 |
|  | Sleep apnea risk | 0.151 | 0.540 | 0.350 |
|  | Healthy sleep pattern | 0.559 | 0.652 | 0.665 |
| **HEI-2015** | Insomnia risk | 0.696 | 0.756 | 0.498 |
|  | Sleep apnea risk | 0.196 | 0.593 | 0.376 |
|  | Healthy sleep pattern | 0.070 | 0.084 | 0.114 |
| **aMed** | Insomnia risk | 0.366 | 0.482 | 0.387 |
|  | Sleep apnea risk | 0.595 | 0.695 | 0.901 |
|  | Healthy sleep pattern | 0.031 | 0.027 | 0.043 |
| **Effect modifier: Race** | | | | |
| **AHEI-2010** | Insomnia risk | 0.087 | 0.023 | 0.023 |
|  | Sleep apnea risk | 0.169 | 0.122 | 0.225 |
|  | Healthy sleep pattern | 0.041 | 0.070 | 0.062 |
| **HEI-2015** | Insomnia risk | 0.149 | 0.152 | 0.310 |
|  | Sleep apnea risk | 0.790 | 0.906 | 0.880 |
|  | Healthy sleep pattern | 0.0007 | <0.0001 | <0.0001 |
| **aMed** | Insomnia risk | 0.177 | 0.011 | 0.167 |
|  | Sleep apnea risk | 0.148 | 0.144 | 0.005 |
|  | Healthy sleep pattern | 0.002 | 0.082 | <0.0001 |
| **Effect modifier: Education** | | | | |
| **AHEI-2010** | Insomnia risk | 0.627 | 0.889 | 0.706 |
|  | Sleep apnea risk | 0.881 | 0.480 | 0.835 |
|  | Healthy sleep pattern | 0.915 | 0.574 | 0.945 |
| **HEI-2015** | Insomnia risk | 0.815 | 0.971 | 0.743 |
|  | Sleep apnea risk | 0.023 | 0.348 | 0.030 |
|  | Healthy sleep pattern | NS | NS | NS |
| **aMed** | Insomnia risk | 0.937 | 0.904 | 0.798 |
|  | Sleep apnea risk | 0.027 | 0.027 | 0.030 |
|  | Healthy sleep pattern | NS | NS | NS |

Table S8. Stratified analyses not reported in main tables, where one or more interaction terms were statistically significant. Risk ratios for sleep outcomes by baseline dietary pattern scores stratified by sex or race.

|  |  | **Quintile of dietary pattern score at baseline (2001-2002)** | | | | | **p for trend** | **per SD increase ^a^** | **p inter-action ^b^** |
| --- | --- | --- | --- | --- | --- | --- | --- | --- | --- |
|  |  | **Q1** | **Q2 ^a^** | **Q3 ^a^** | **Q4 ^a^** | **Q5 ^a^** |  |  |  |
| **Sex stratified** (n men = 215, n women = 378) | | | | | | | | | |
| **aMed** | |  |  |  |  |  |  |  |  |
| **Healthy sleep** | men | 1.00 | 0.81 (0.52, 1.26) | 0.95 (0.47, 1.93) | 1.06 (0.48, 2.37) | 1.42 (0.67, 3.05) | 0.330 | 1.14 (0.88, 1.47) | 0.031; 0.027; 0.043 |
|  | women | 1.00 | 1.15 (0.70, 1.92) | 0.84 (0.57, 1.23) | 0.64 (0.43, 0.95) | 0.83 (0.55, 1.27) | 0.100 | 0.87 (0.75, 1.02) |  |
| **Race stratified** (n white = 415, n black = 178) | | | | | | | | | |
| **AHEI** | |  |  |  |  |  |  |  |  |
| **Healthy sleep** | Black | 1.00 | *Model not able to converge* | | | | | | 0.041; 0.070; 0.062 |
|  | White | 1.00 | 1.12 (0.71, 1.77) | 0.98 (0.61, 1.57) | 1.30 (0.83, 2.03) | 1.75 (0.99, 3.09) ^*^ | 0.039 | 1.16 (1.00, 1.35) |  |
| **HEI** | |  |  |  |  |  |  |  |  |
| **Healthy sleep** | Black | 1.00 | *Model not able to converge* | | | | | | 0.0007; <0.0001; <0.0001 |
|  | White | 1.00 | 1.77 (0.78, 4.00) | 1.15 (0.64, 2.05) | 1.80 (1.06, 3.06) ^*^ | 1.51 (0.79, 2.89) | 0.125 | 1.12 (0.96, 1.29) |  |
| **aMed** | |  |  |  |  |  |  |  |  |
| **Insomnia risk** | Black | 1.00 | 1.13 (0.62, 2.07) | 1.37 (0.80, 2.33) | 1.16 (0.67, 1.99) | 1.55 (0.96, 1.49) | 0.055 | 1.10 (0.96, 1.26) | 0.177; 0.011; 0.167 |
|  | White | 1.00 | 0.71 (0.54, 0.93) ^*^ | 0.75 (0.63, 0.89) ^**^ | 0.80 (0.57, 1.13) | 0.83 (0.60, 1.15) | 0.435 | 0.95 (0.85, 1.06) |  |
| **Sleep apnea risk** | Black | 1.00 | 0.95 (0.33, 2.75) | 1.77 (0.97, 3.21) | 1.63 (1.02, 2.60) ^*^ | 1.60 (0.80, 3.20) | 0.037 | 1.13 (0.92, 1.39) | 0.148; 0.144; 0.005 |
|  | White | 1.00 | 1.18 (0.82, 1.69) | 0.99 (0.69, 1.42) | 1.04 (0.73, 1.50) | 0.81 (0.61, 1.08) | 0.119 | 0.94 (0.85, 1.02) |  |
| **Healthy sleep** | Black | 1.00 | *Model not able to converge* | | | | | | 0.002; 0.082; <0.0001 |
|  | White | 1.00 | 0.91 (0.50, 1.64) | 1.08 (0.73, 1.62) | 0.86 (0.58, 1.27) | 1.35 (0.87, 2.10) | 0.236 | 1.11 (0.95, 1.30) |  |
| ^a.^ Risk Ratio (95% Confidence Interval)  ^b.^ p for interaction between the effect modifier of interest (sex, race, or education) and three versions of dietary pattern: continuous; Q5 (vs. Q1); and trend variable  ^*^ p < 0.05, ^**^ p < 0.01, ^***^ p < 0.001  Models adjusted for: total energy intake, age, sex, race, education (any college or higher), employed (full or part time), income category, number of people in house, spouse lives in house, total population of census tract, ICE of census tract, smoking status (never, current, former), drinking status (non-, occasional, regular drinker), caffeine intake (mg/d), depressive symptoms (CES-D≥16), body mass index (kg/m^2^), physical active when not at work (4 or 5 [active or very active] on 5-point self-rating scale). | | | | | | | | | |

Table S9. Sensitivity analysis adjusting for sleep duration: Risk ratios for high insomnia symptoms at follow-up by baseline dietary pattern scores (n-571).

|  | **Quintile of dietary pattern score at baseline (2001-2002)** | | | | | **p for trend** | **per SD increase ^a^** |
| --- | --- | --- | --- | --- | --- | --- | --- |
|  | **Q1** | **Q2 ^a^** | **Q3 ^a^** | **Q4 ^a^** | **Q5 ^a^** |  |  |
| AHEI-2010 | 1.00 | 0.76 (0.58, 0.99) ^*^ | 0.83 (0.67, 1.04) | 0.78 (0.60, 0.99) ^*^ | 0.53 (0.38, 0.73) ^***^ | 0.0002 ^***^ | 0.84 (0.77, 0.93) ^***^ |
| HEI-2015 | 1.00 | 1.02 (0.80, 1.29) | 0.79 (0.61, 1.03) | 0.66 (0.50, 0.87) ^**^ | 0.67 (0.50, 0.90 ^**^ | 0.0003 ^***^ | 0.86 (0.78, 0.94 ^**^ |
| aMed | 1.00 | 0.79 (0.58, 1.06) | 0.83 (0.63, 1.07) | 0.77 (0.57, 1.04) | 0.89 (0.69, 1.16) | 0.540 | 0.97 (0.88, 1.06) |

^a.^ Risk Ratio (95% Confidence Interval) for having high risk of insomnia based on the Women’s Health Initiative Insomnia Rating Scale)

^*^ p < 0.05, ^**^ p < 0.01, ^***^ p < 0.001

AHEI: Alternate Healthy Eating Index

HEI: Healthy Eating Index

aMed: Alternate Mediterranean dietary pattern

Model included the following variables: total energy intake, age, sex, race, education (any college or higher), employed (full or part time), income category, number of people in house, spouse lives in house, total population of census tract, ICE of census tract, smoking status (never, current, former), drinking status (non-, occasional, regular drinker), caffeine intake (mg/d), depressive symptoms (CES-D≥16), body mass index (kg/m^2^), physical active when not at work (4 or 5 [active or very active] on 5-point self-rating scale), sleep duration at follow-up (weighted average of self-reported usual nightly sleep duration on weekdays and weekends).

Table S10. Sensitivity analysis removing BMI from models for sleep apnea: Risk ratios for high sleep apnea risk at follow-up by baseline dietary pattern scores.

|  | **Quintile of dietary pattern score at baseline (2001-2002)** | | | | | **p for trend** | **per SD increase ^a^** |
| --- | --- | --- | --- | --- | --- | --- | --- |
|  | **Q1** | **Q2 ^a^** | **Q3 ^a^** | **Q4 ^a^** | **Q5 ^a^** |  |  |
| AHEI-2010 | 1.00 | 0.84 (0.63, 1.12) | 0.83 (0.62, 1.10) | 0.85 (0.63, 1.14) | 0.68 (0.48, 0.97) ^*^ | 0.051 | 0.91 (0.81, 1.01) |
| HEI-2015 | 1.00 | 1.15 (0.84, 1.58) | 1.27 (0.93, 1.73) | 1.10 (0.79, 1.52) | 0.90 (0.62, 1.31) | 0.460 | 0.93 (0.84, 1.03) |
| aMed | 1.00 | 0.97 (0.68, 1.40) | 1.06 (0.77, 1.48) | 1.11 (0.81, 1.52) | 0.93 (0.66, 1.30) | 0.833 | 0.98 (0.88, 1.09) |

^a.^ Risk Ratio (95% Confidence Interval) for having high sleep apnea risk based on the Berlin Questionnaire

^*^ p < 0.05, ^**^ p < 0.01, ^***^ p < 0.001

AHEI: Alternate Healthy Eating Index

Model included the following variables: total energy intake, age, sex, race, education (any college or higher), employed (full or part time), income category, number of people in house, spouse lives in house, total population of census tract, ICE of census tract, smoking status (never, current, former), drinking status (non-, occasional, regular drinker), caffeine intake (mg/d), depressive symptoms (CES-D≥16), physical active when not at work (4 or 5 [active or very active] on 5-point self-rating scale).

Table S11. Risk ratios for sleep outcomes, components of the Berlin Questionnaire, at follow-up by baseline dietary pattern scores.

|  | **Quintile of dietary pattern score at baseline (2001-2002)** | | | | | **p for trend** | **per SD increase ^a^** |
| --- | --- | --- | --- | --- | --- | --- | --- |
|  | **Q1** | **Q2 ^a^** | **Q3 ^a^** | **Q4 ^a^** | **Q5 ^a^** |  |  |
| **AHEI 2010** | n=127 | n=116 | n=129 | n=112 | n=109 |  | SD=7.8 |
| Berlin Cat 1: Snoring | 1.00 | 0.88 (0.68, 1.15) | 0.84 (0.64, 1.11) | 0.86 (0.66, 1.14) | 0.67 (0.48, 0.92) ^*^ | 0.017 ^*^ | 0.89 (0.81, 0.98) ^*^ |
| Berlin Cat 2: Sleepiness | 1.00 | 0.84 (0.54, 1.30) | 0.85 (0.54, 1.33) | 0.64 (0.40, 1.05) | 0.51 (0.28, 0.94) ^*^ | 0.014 ^*^ | 0.80 (0.67, 0.96) ^*^ |

^a.^ Risk Ratio (95% Confidence Interval)

^*^ p < 0.05, ^**^ p < 0.01, ^***^ p < 0.001

AHEI: Alternate Healthy Eating Index

Model 3 shown, included the following variables: total energy intake, age, sex, race, education (any college or higher), employed (full or part time), income category, number of people in house, spouse lives in house, total population of census tract, ICE of census tract, smoking status (never, current, former), drinking status (non-, occasional, regular drinker), caffeine intake (mg/d), depressive symptoms (CES-D≥16), body mass index (kg/m^2^), physical active when not at work (4 or 5 [active or very active] on 5-point self-rating scale).

Table S12. Risk ratios for being healthy on components of the healthy sleep pattern, at follow-up by baseline dietary pattern scores.

|  | **Quintile of dietary pattern score at baseline (2001-2002)** | | | | | **p for trend** | **per SD increase ^a^** |
| --- | --- | --- | --- | --- | --- | --- | --- |
|  | **Q1** | **Q2 ^a^** | **Q3 ^a^** | **Q4 ^a^** | **Q5 ^a^** |  |  |
| **AHEI 2010** | n=127 | n=116 | n=129 | n=112 | n=109 |  | SD=7.8 |
| HSP 1: Chronotype | 1.00 | 1.22 (0.94, 1.58) | 1.21 (0.93, 1.58) | 1.32 (1.01, 1.73) | 1.13 (0.85, 1.50) | 0.376 | 1.04 (0.96, 1.13) |
| HSP 2: Duration | 1.00 | 0.95 (0.74, 1.20) | 1.04 (0.83, 1.29) | 1.03 (0.82, 1.30) | 1.00 (0.78, 1.29) | 0.787 | 1.01 (0.93, 1.09) |
| HSP 3: Insomnia | 1.00 | 1.03 (0.68, 1.54) | 0.64 (1.01, 0.05) | 1.22 (0.81, 1.83) | 1.12 (0.70, 1.81) | 0.469 | 1.01 (0.86, 1.18) |
| HSP 4: Snoring | 1.00 | 1.28 (0.96, 1.70) | 1.29 (0.97, 1.72) | 1.29 (0.96, 1.73) | 1.54 (1.15, 2.07) ^**^ | 0.007 ^**^ | 1.13 (1.03, 1.23) ^**^ |
| HSP 5: Sleepiness | 1.00 | 1.06 (0.97, 1.16) | 0.99 (0.90, 1.16) | 0.97 (0.87, 1.08) | 1.05 (0.94, 1.17) | 0.818 | 1.01 (0.98, 1.04) |

^a.^ Risk Ratio (95% Confidence Interval) for scoring as healthy on each sleep component

^*^ p < 0.05, ^**^ p < 0.01, ^***^ p < 0.001

AHEI: Alternate Healthy Eating Index

HSP 1-Chronotype: healthy if “morning type” or “more morning than evening type” based on the reduced Morningness-Eveningness Questionnaire.

HSP 2-Duration: healthy if average self-report sleep duration ≥7 hours and <9 hours.

HSP 3-Insomnia symptoms: healthy if no frequent insomnia symptoms, from the Women’s Health Initiative Insomnia Rating Scale.

HSP 4-Snoring: healthy if no snoring in the past 4 weeks, from the Berlin Questionnaire.

HSP 5-Sleepiness: healthy if score <11 on the Epworth Sleepiness Scale.

Model 3 shown, included the following variables: total energy intake, age, sex, race, education (any college or higher), employed (full or part time), income category, number of people in house, spouse lives in house, total population of census tract, ICE of census tract, smoking status (never, current, former), drinking status (non-, occasional, regular drinker), caffeine intake (mg/d), depressive symptoms (CES-D≥16), body mass index (kg/m^2^), physical active when not at work (4 or 5 [active or very active] on 5-point self-rating scale).
